# Tumor Thrombus of Hepatocellular Carcinoma: A Direct Extension From the Liver to the Right Atrium

**DOI:** 10.7759/cureus.55158

**Published:** 2024-02-28

**Authors:** Moutaz Ghrewati, Anas Mahmoud, Tala Beilani, Sean A Keegan, Mehandar Kumar

**Affiliations:** 1 Oncology, St. Joseph’s University Medical Center, Paterson, USA; 2 Internal Medicine, St. Joseph’s University Medical Center, Paterson, USA; 3 Pathology, St. Joseph’s University Medical Center, Paterson, USA

**Keywords:** hepatocellular carcinoma (hcc), ivc thrombus, milan criteria, radio-embolization, right atrium tumor thrombus

## Abstract

Hepatocellular carcinoma (HCC) is a very aggressive type of cancer and can either invade or spread distantly through the portal vein to the inferior vena cava (IVC) and the right atrium (RA). The presentation varies based on the stage of the cancer at the time of diagnosis. Liver transplantation or surgical resection is the ideal management of small lesions without metastases, while systemic therapy can help in extensive cases to decrease the tumor burden to allow surgical resection of the tumor. We present a rare case of HCC with a tumor thrombus (TT) extending to the RA. Unfortunately, the patient did not survive the cancer. We hope that this case report can contribute to saving the lives of future patients with HCC.

## Introduction

Hepatocellular carcinoma (HCC) displays a vascular invasion pattern through the formation of a tumor thrombus (TT) and tends to extend into the portal vein. Rarely, HCC can extend to the hepatic vein (HV), inferior vena cava (IVC), and right atrium (RA) [1]. If that happens, the prognosis is dramatically dismal. The symptoms depend on the extent of the vein occlusion caused by the TT. In incomplete blockage cases, no special manifestation occurs; however, if the occlusion is complete, characteristics of Budd-Chiari syndrome ensue, such as ascites, esophageal or gastric varices, pleural effusion, or lower extremity edema [2]. Severe cases can present with pulmonary embolism and sudden cardiac death.

The Milan criteria can guide the management of HCC in its early stages [3]. However, in the instance of the extremely rare TT extension to RA, there is no universally agreeable plan. The prognosis for metastatic HCC is bleak, and in cases of RA extension of the TT, options are very limited, leaving only comfort measures [3]. We present a rare case of HCC that presented as a TT extending from the liver to the RA. The patient was planned to receive radioembolization; however, he did not follow up, and unfortunately, he presented later in poor condition where comfort measures were provided, and he eventually passed away. 

## Case presentation

**Figure 1:**
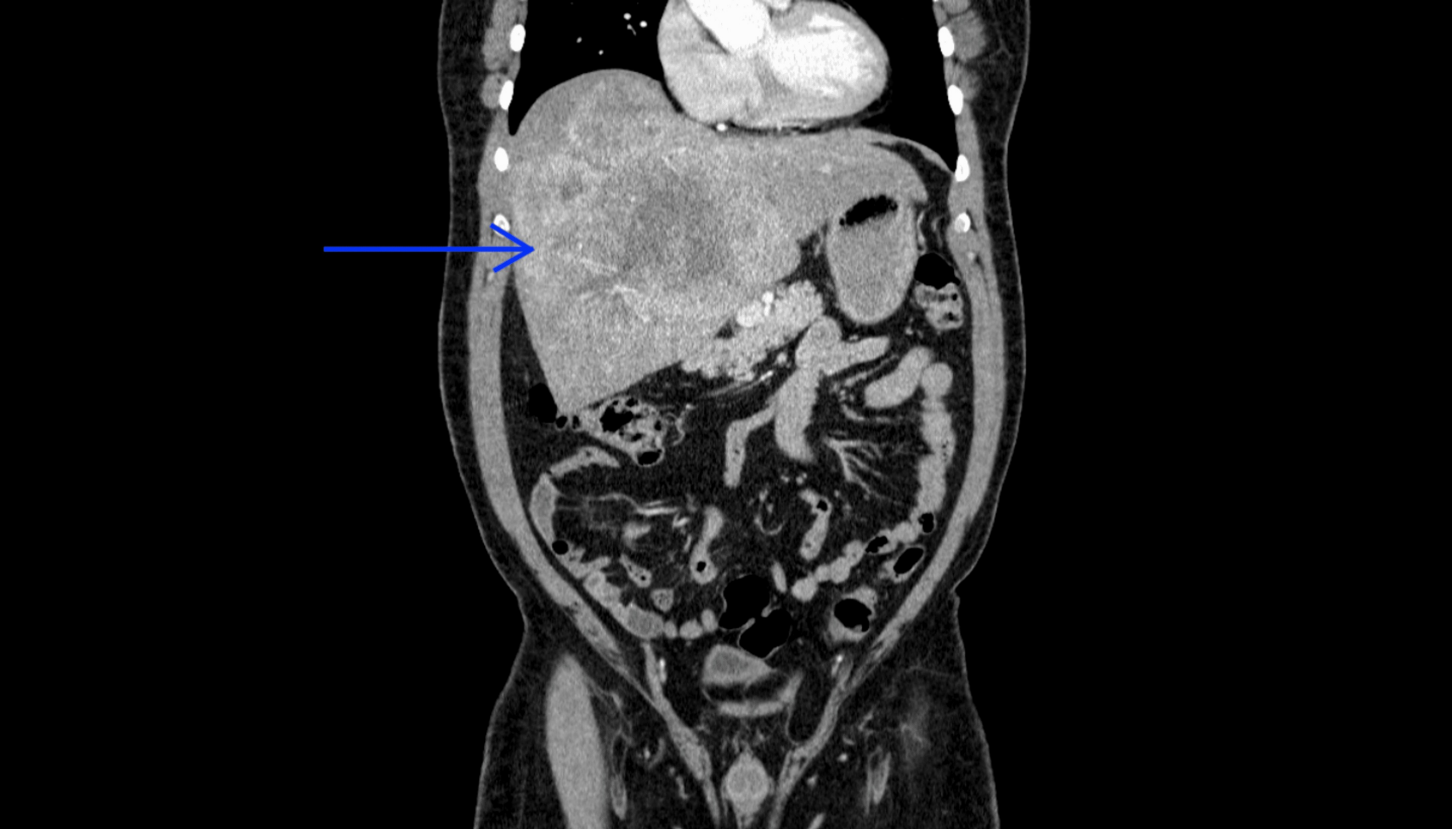
The CT of the abdomen with contrast Seen is a very large heterogeneously enhancing mass centered in the right hepatic lobe, measuring 18.3 cm x 11 cm (blue arrow).

The patient was a 51-year-old male with a past medical history of hypertension, cerebrovascular stroke, and alcohol abuse who presented to the ER with complaints of right-sided abdominal pain radiating to the back and left upper quadrant abdomen. The pain started two months prior to presentation, and since then, he lost 15 pounds unintentionally. The patient elaborated that he used to drink alcohol heavily for years and quit drinking six months before presentation. He also reported that he used to smoke tobacco many years ago and had not smoked for 10 years. On examination, the abdomen was tender to palpation. A CT scan of the abdomen with contrast showed a very large, heterogeneously enhancing mass centered in the right hepatic lobe, measuring 18.3 cm x 11 cm, compressing the IVC (Figure 1).

**Figure 2:**
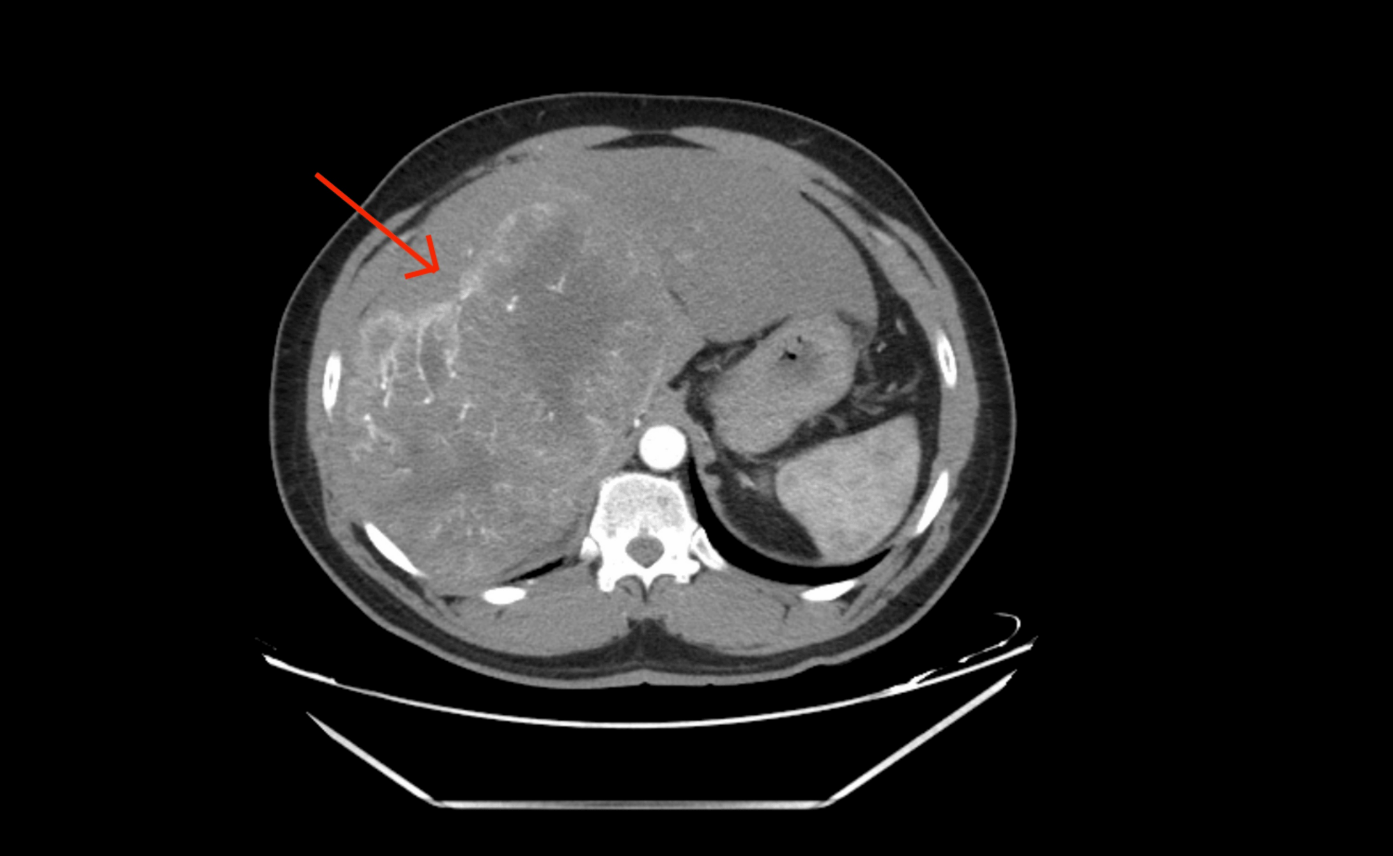
Triple-phase CT with liver mass protocol (arterial phase) The enhanced mass is seen with an enhancing capsule around it, compared to the rest of the liver tissue. This is because the mass is getting blood supply from the hepatic artery (early enhancement), while the rest of the liver is receiving it from the portal vein.

**Figure 3:**
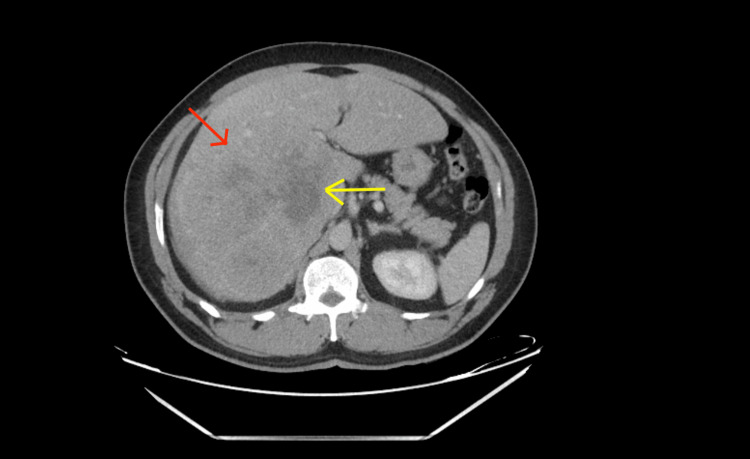
Triple-phase CT with liver mass protocol (venous phase) A decreased enhancement of the capsule around the mass (red arrow) is seen with a further low enhancement of the mass (yellow arrow) as the rest of the liver is receiving blood supply containing the contrast from the portal vein.

**Figure 4:**
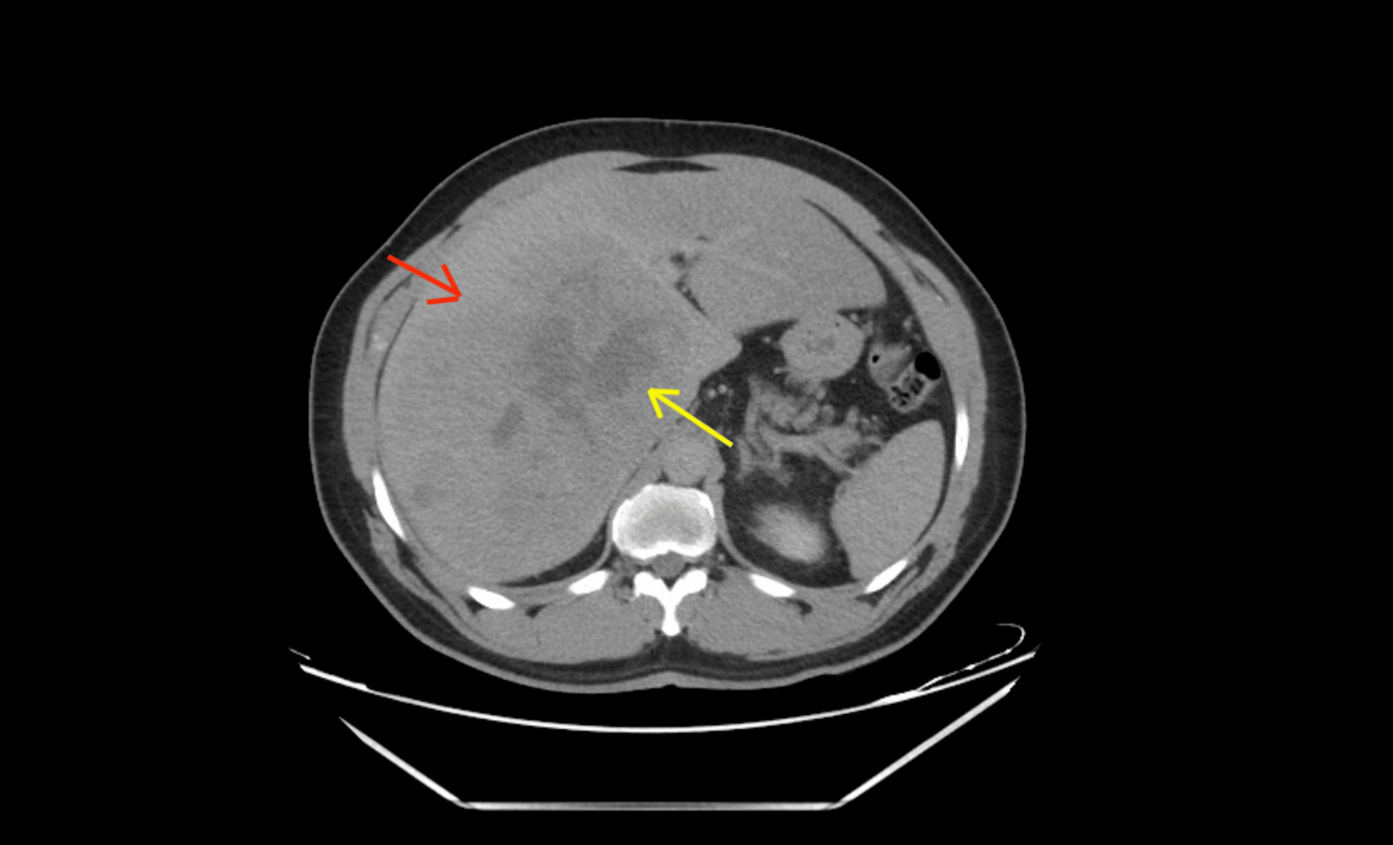
Triple-phase CT with liver mass protocol (delayed phase) Seen is a wash of the enhancement of the mass and the capsule around the mass (red arrow) and further hypodensity of the mass (yellow arrow). This is evident as the rest of the liver is getting more blood supply containing the contrast from the portal vein compared to the mass, which is receiving its supply from the hepatic artery.

Blood work was significant for elevated aspartate aminotransferase (AST), alanine transaminase (ALT), total bilirubin, and alkaline phosphatase (ALP). The hepatitis panel came back negative for hepatitis B virus (HBV) and hepatitis C virus (HCV). The alpha-fetoprotein (AFP) level was more than 15,000. Triple-phase CT abdomen and pelvis showed an arterially enhancing mass occupying the majority of the right hepatic lobe, measuring 18 cm x 11 cm, and demonstrating diffuse peripheral arterial enhancement. There were also more central areas of the mass that did not demonstrate enhancement, indicating a large necrotic HCC, particularly in light of elevated AFP (Figures 2-4).

**Figure 5:**
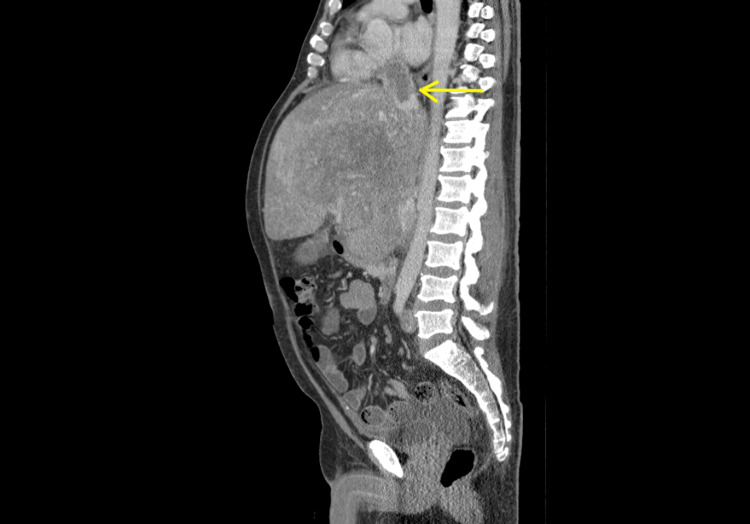
Sagittal view of the repeat CT of chest and abdomen with contrast Seen is the extension of the tumor to the IVC and the RA (yellow arrow). IVC: Inferior vena cava, RA: Right atrium

**Figure 6:**
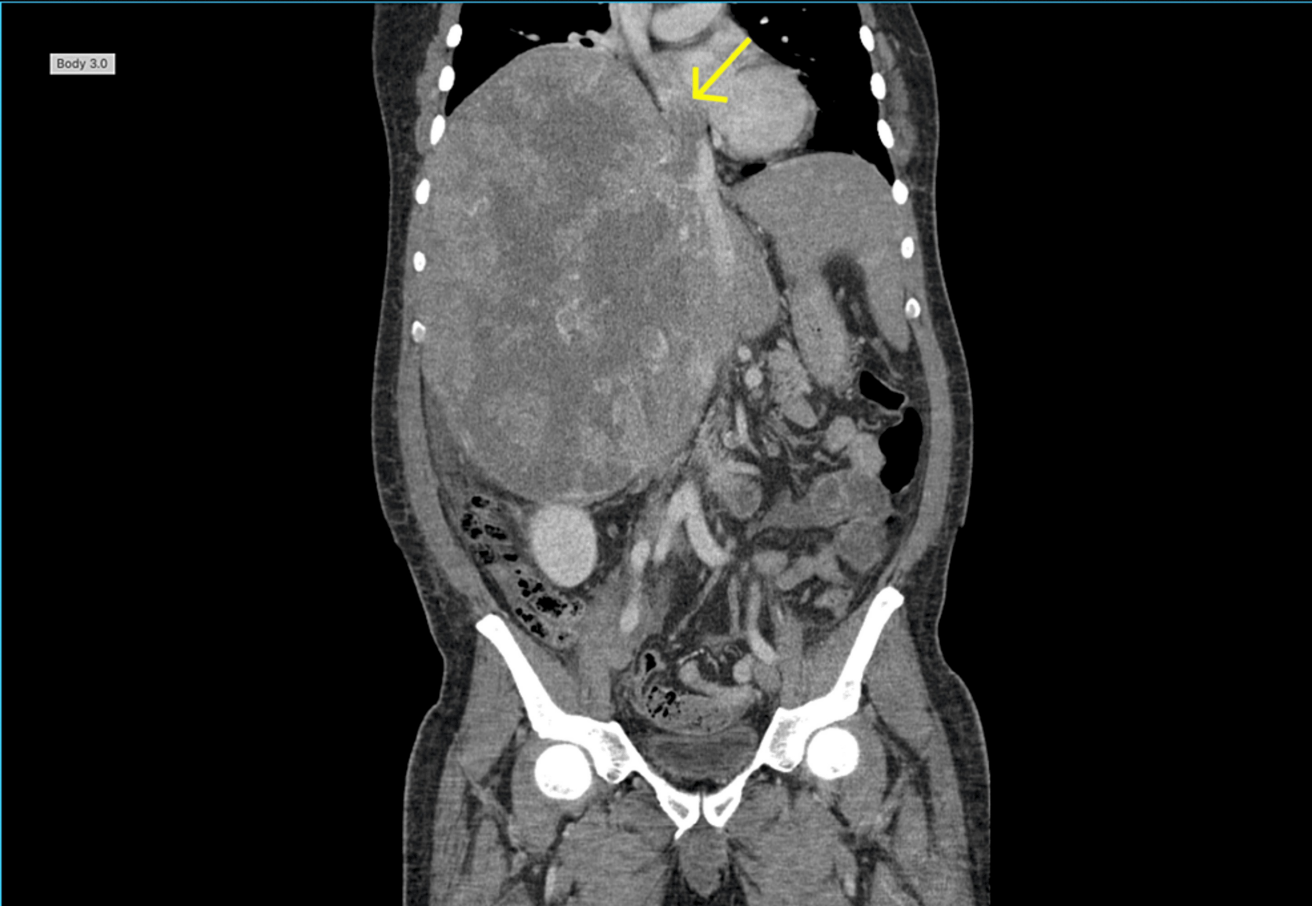
Coronal view of the repeat CT chest and abdomen with contrast Displayed is the extension of the tumor to the IVC and the RA (yellow arrow). IVC: Inferior vena cava, RA: Right atrium

The CT scan of the chest and the bone scan were negative for any metastatic lesions. A liver biopsy showed moderately differentiated HCC. The case was discussed with a multidisciplinary team, and the patient was deemed to be a candidate for Y-90 radioembolization as an outpatient. Unfortunately, due to socioeconomic circumstances, the patient was lost to follow-up until he was admitted two months later for intractable abdominal pain with cachexia and a significant weight loss of 30 lbs. Repeat imaging CT scans of the abdomen and pelvis with contrast revealed an interval increase in the size of the liver mass to 29 cm x 26 cm and extension into the IVC and RA (Figures 5-6).

The case was discussed again with the multidisciplinary team. A plan was made to proceed with palliative radiation therapy to control the symptoms and alleviate the compression of the major vessels, and subsequently treat the patient with nivolumab given his borderline Eastern Cooperative Oncology Group (ECOG) score of 3 and Child-Pugh score of B7. The patient then received one session of palliative 8GY radiotherapy to control symptoms. However, prior to receiving nivolumab, the patient unfortunately presented with severe hepatic encephalopathy. Lamentably, all supportive measures failed to improve the patient's condition, and he gradually deteriorated. Hence, the goals of care were switched to focus on his comfort, and he passed away a few days later. 

## Discussion

Hepatocellular carcinoma is the fifth most common cancer worldwide and the second most common cause of cancer-related death worldwide [1]. Risk factors for developing HCC include cirrhosis, alcohol abuse, smoking, hepatitis B and C virus infection, etc. Nearly 80% of HCC patients have underlying cirrhosis [1]. Hence, all cirrhotic patients, regardless of the cause, should be screened with abdomen ultrasonography every six months and measured for AFP. The HBV vaccine is the most important preventive measure for HCC because HBV can bypass cirrhosis and also result suddenly in cirrhosis; hence, all chronically HBV-infected patients should undergo screening as well. The diagnosis of HCC is usually made by a typical imaging finding causing arterial enhancement and delayed washout [2]. The hepatic artery is the main feeder of the HCC; hence, the avid contrast enhancement during the arterial phase during imaging can detect very small lesions <1 cm. A portal-delayed phase washout is characteristic of HCC [2]. These typical findings can eliminate the need for a biopsy to confirm the diagnosis of HCC. Imaging would reveal either a few large lesions or multiple small lesions, but to our knowledge, not many cases showed this extensive expansion of the lesion [4,5].

Treatment of HCC is quite challenging as it depends on many factors, including, but not limited to, the patient’s performance status to determine the safety of the operation, the size, and number of lesions, the proximity to major vessels to identify the resectability of the tumor, and the baseline function evaluated by the Child-Pugh score. Therefore, the decision should be made after a multidisciplinary team discussion. Surgical resection is the mainstay of the treatment for localized HCC. The Milan criteria are used to manage invaded HCC: a single tumor diameter less than 5 cm; not more than three foci of tumor, each one not exceeding 3 cm; no angioinvasion; and no extrahepatic involvement.

Xia et al. [3] published a detailed review of diagnosis, treatment, and prognosis in cases where TT extends to the IVC and RA. Tumor thrombus occurs more commonly in the portal vein system, with an incidence reaching up to 62% of all HCC cases [4,6], and is rarely found in the HV, IVC, or RA, with an incidence of only 1.4% to 4.9% [7]. Unfortunately, the prognosis becomes extremely poor, and the median survival of untreated patients ranges between two and five months. Tumor thrombus in the RA more commonly occurs as an extension of TT from the IVC than in isolation. In type 1 TT, the IVC thrombus does not exceed the diaphragm; in type II, the IVC TT exceeds the diaphragm but not the heart; and in type III, the thrombus extends into the RA [7]. In type III, both cardiothoracic and hepatobiliary surgery should include hepatectomy and RA TT resection, requiring surgery under extracorporeal circulation to bypass the blood flow from the superior vena cava and IVC to the ascending aorta after oxygenation to allow incision of the RA and excision of TT en bloc under direct vision [4].

Given the high risk of surgery, only patients with Child-Pugh class A can qualify. Postoperative sorafenib and adjuvant transarterial chemoembolization (TACE) are recommended to prevent recurrence [8]. The efficacy of radiation therapy in TT extension to IVC or RA could offer a significant survival advantage compared to surgical resection.

## Conclusions

Hepatocellular carcinoma with TT extension has a very poor prognosis as it is a very late stage of cancer presentation. As the resection of the thrombus is the definitive management, locoregional therapy, along with other modalities, can help shrink the cancer. However, this necessitates good functional status, which is usually lacking in these late stages of HCC. New trials of TACE plus systemic chemotherapy or radiation therapy have shown promising results in HCC with TT extension, offering hope to patients.

## References

[1] Chidambaranathan-Reghupaty S, Fisher PB (2020). Hepatocellular carcinoma (HCC): epidemiology, etiology, and molecular classification. Adv Cancer Res.

[2] Shah S, Shukla A, Paunipagar B (2014). Radiological features of hepatocellular carcinoma. J Clin Exp Hepatol.

[3] Xia Y, Zhang J, Ni X (2020). Diagnosis, treatment and prognosis of hepatocellular carcinoma with inferior vena cava/right atrium tumor thrombus. Oncol Lett.

[4] Sugawara Y, Hibi T (2021). Surgical treatment of hepatocellular carcinoma. Biosci Trends.

[5] Morrell GR (2022). Detection of tumor thrombus in hepatocellular carcinoma. Radiology.

[6] Khan AR, Wei X, Xu X (2021). Portal vein tumor thrombosis and hepatocellular carcinoma — the changing tides. J Hepatocell Carcinoma.

[7] Liu J, Zhang RX, Dong B, Guo K, Gao ZM, Wang LM (2021). Hepatocellular carcinoma with inferior vena cava and right atrium thrombus: a case report. World J Clin Cases.

[8] Chang Y, Jeong SW, Jang JY, Jae Kim Y (2020). Recent updates of transarterial chemoembolilzation in hepatocellular carcinoma. Int J Mol Sci.

